# Persistent and transient olfactory deficits in COVID-19 are associated to inflammation and zinc homeostasis

**DOI:** 10.3389/fimmu.2023.1148595

**Published:** 2023-07-14

**Authors:** Lorenzo Lupi, Anna Bordin, Gabriele Sales, Davide Colaianni, Adriana Vitiello, Alberto Biscontin, Alberto Reale, Alfredo Garzino-Demo, Angelo Antonini, Giancarlo Ottaviano, Carla Mucignat, Cristina Parolin, Arianna Calistri, Cristiano De Pittà

**Affiliations:** ^1^ Department of Biology, University of Padova, Padova, Italy; ^2^ Department of Molecular Medicine, University of Padova, Padova, Italy; ^3^ Department of Neurosciences, Otolaryngology Section, University of Padova, Padova, Italy; ^4^ Department of Microbiology and Immunology, School of Medicine, University of Maryland, Baltimore, Maryland, United States; ^5^ Parkinson and Movement Disorders Unit, Department of Neurosciences, University of Padova, Padova, Italy; ^6^ Department of Neurosciences, University of Padova, Padova, Italy

**Keywords:** SARS-CoV-2, olfactory symptoms, RNA-seq, miRNA, inflammation, zinc homeostasis, metallothionein

## Abstract

**Introduction:**

The Coronavirus Disease 2019 (COVID-19) is mainly a respiratory syndrome that can affect multiple organ systems, causing a variety of symptoms. Among the most common and characteristic symptoms are deficits in smell and taste perception, which may last for weeks/months after COVID-19 diagnosis owing to mechanisms that are not fully elucidated.

**Methods:**

In order to identify the determinants of olfactory symptom persistence, we obtained olfactory mucosa (OM) from 21 subjects, grouped according to clinical criteria: i) with persistent olfactory symptoms; ii) with transient olfactory symptoms; iii) without olfactory symptoms; and iv) non-COVID-19 controls. Cells from the olfactory mucosa were harvested for transcriptome analyses.

**Results and discussion:**

RNA-Seq assays showed that gene expression levels are altered for a long time after infection. The expression profile of micro RNAs appeared significantly altered after infection, but no relationship with olfactory symptoms was found. On the other hand, patients with persistent olfactory deficits displayed increased levels of expression of genes involved in the inflammatory response and zinc homeostasis, suggesting an association with persistent or transient olfactory deficits in individuals who experienced *SARS-CoV-2* infection.

## 1 Introduction

At the end of 2019 a new coronavirus, subsequently named Severe Acute Respiratory Syndrome Coronavirus 2 (*SARS-CoV-2*), emerged in the human population in the city of Wuhan, China, rapidly spreading worldwide and causing a spectrum of pathological manifestations known as Coronavirus Disease 2019 (COVID-19) (1, 2). Although, COVID-19 is mainly a respiratory disease, additional organs, and systems, such as liver and brain, may be affected (3, 4). In particular, since the beginning of the pandemic, deficits in olfactory and taste perception possibly leading to their complete loss (termed anosmia and ageusia, respectively) appeared as common and characteristic early symptoms in a relevant percentage of *SARS-CoV-2* infected patients (5–9). While anosmia and ageusia are typical manifestations of different upper respiratory tract infections (10), in the case of COVID-19 they are not necessarily associated to mucosal congestion (11, 12). Although most cases of *SARS-CoV-2* related anosmia are transient, the recovery from this condition was found to be highly variable among individuals. Both the mechanisms implicated in loss of smell and the reasons accounting for its variable duration have not been fully elucidated yet. A recent multi-ancestry genome-wide association study provided the first genetic link to the biological mechanisms underlying COVID-19-related chemosensory deficits (13). This work identified a genome-wide significant locus in the proximity of two genes, *UGT2A1* and *UGT2A2*, which play a role in metabolizing odorants. To cause anosmia, *SARS-CoV-2* must directly and/or indirectly interact with tissues and factors contributing to the recognition of odorants, such as the olfactory mucosa (OM). The OM, which is located in the upper and deeper part of the nasal cavity including also part of the nasal septum (14) is constituted by a complex network of neural and non-neuronal cells distributed in two layers: the lamina propria and the olfactory neuroepithelium. The latter encompasses olfactory sensory neurons (OSN), non-neuronal supporting sustentacular cells (SuCs), as well as Bowman glands and basal cells. In the surrounding lamina propria olfactory ensheathing cells (OECs), that are involved in neural regeneration, can be found along with fibroblasts and mesenchymal stem cells (14–16). Early works suggested a viral mediated destruction of OSNs as the main cause of SARS-CoV-2 related anosmia (17, 18). However, patients-based studies did not fully support this conclusion (19). As SuCs express both the angiotensin-converting enzyme 2 (ACE-2) and the transmembrane serine protease TMPRSS (16, 17), which are crucial for *SARS-CoV-2* entry into target cells (20), studies focused on this cellular type. Subsequent studies showed that SuCs can be indeed infected by the virus in an animal model (21). Death of SuCs combined with the effect of the local immune response and inflammation could result in the OM damage that leads to anosmia (17). Nonetheless, it is not clear whether these findings apply to the clinical course of COVID-19. Interestingly neuropilin-1 (NRP-1), an additional protein involved in *SARS-CoV-2* entry into target cells, was found to be highly expressed in the olfactory and respiratory epithelia (22). Importantly, NRP-1 is involved in the olfactory axonal guidance as it interacts with Semaphorin-3A (SEMA3A) a protein crucial in this process (23, 24). However, *SARS-CoV-2* cannot infect cells by binding NRP-1 alone in the absence of ACE2 co-expression so the NRP-1 hypothesis has not been supported by data. More recently, it has been proposed that *SARS-CoV-2* related anosmia could be caused by all of the above-described mechanisms (direct and indirect damage of OSNs and SuCs, inhibition of NRP-1/SEMA3A signaling pathway, local immune response and inflammation), resulting in a global downregulation of olfactory receptors and signaling pathways (25). While some of the patients experience a full recovery in a few days/weeks upon infection resolution, others continue to suffer from olfactory deficits for months (26). Among the hypotheses explaining this prolonged loss of smell there are, i) the persistence of *SARS CoV-2* in the OM (27), ii) the lack of recovery of the viral damaged cells, iii) a chronic inflammation of the OM. In order to gain insight on the molecular mechanisms accounting for loss of smell and with its persistence in *SARS-CoV-2* patients, we enrolled a cohort of individuals i) who was still experiencing smell dysfunction up to 4 months post resolution of the infection, ii) who had recovered from COVID-19 smell dysfunction, iii) who had experienced COVID-19 with no olfactory symptoms, iv) who never tested positive for *SARS-CoV-2*. By adopting microarray and RNA-Seq techniques for the whole transcriptome analysis here we demonstrate that i) miRNAs do not correlate with the severity of symptoms as they are not differentially expressed in patients with persistent *versus* patients with no olfactory symptoms, ii) gene expression signatures discriminate controls from *SARS-CoV-2* patients and the latter in groups according to the displayed olfactory symptoms; iii) among differentially expressed genes, some encoding metallothioneins involved in zinc homeostasis showed the highest expression levels in the group of patients with persistent anosmia. Overall, our data support the notation that zinc levels and Th2 immune response might play a role in the persistence of olfactory deficits in patients who have experienced *SARS-CoV-2* infection.

## 2 Materials and methods

### 2.1 Patients and samples

Twenty-one subjects were enrolled in the Neurocovid project, conducted at the University of Padova, with the aim of investigating potential *SARS-CoV-2* related transcriptional signatures in olfactory mucosa that might be linked to anosmia. Before entering the study, approved by the local Ethical Committee, all subjects signed a written informed consent form. The enrolled subjects had a mean age of 34 years old, both sexes and, in most cases, without significant lung involvement. In many of them, anosmia or hyposmia persisted at least 4-6 weeks after COVID-19 diagnosis. Following an anamnestic interview, all the patients were assessed using the Sniffin’ stick test (Burghart Medical Technology, Wedel, Germany). The Sniffin’s stick test is a psychophysical validated smell test consisting of three subtests, which allow the study of odor threshold, discrimination and identification, respectively. The sum of the three subtest results gives a composite score, known as TDI (threshold, discrimination, identification), which can indicate normal olfactory function, hyposmia, or anosmia (28) test scores range from 0 to 45, hyposmia (decreased olfactory function) is defined with a score 16<TDI<30, while a TDI score <16 is defined as anosmia (loss of olfactory function). Olfactory epithelium cells were sampled from each subject by using Copan 491CE.A swab (Copan Italy, Brescia, BS, Italy) under endoscopic guide at the University Hospital in Padova. Immediately after sampling, the swab was inserted in RNA/DNA protection buffer and placed on ice (**Supplementary Figure 1**).

### 2.2 Total RNA extraction

Total RNA, including miRNAs, was extracted from olfactory epithelium cells by using Monarch^®^ Total RNA Miniprep kit (New England Biolabs, Ipswich, MA, USA) according to the manufacturer’s instructions. Total RNA was quantified by NanoDrop 2000c (Thermo Fisher Scientific, Waltham, MA, USA). RNA integrity and content of miRNAs (%) in each sample were assessed by capillary electrophoresis with the RNA 6000 Nano LabChip and the Small RNA Nano LabChip, respectively, by using the Agilent Bioanalyzer 2100 (Agilent Technologies, Santa Clara, CA, USA). Both nostrils were sampled independently, and the obtained cells were used for total RNA extraction (**Supplementary Figure 1**). For each subject, only the best sample in terms of RNA quantity and quality was selected and used for further analysis. More detailed information about RNA samples is reported in **Table 1**. Only samples with RNA Integrity Number (R.I.N.) values higher than six and a percentage of miRNA, calculated on the total of small RNA, between 40% and 65% were used for gene and miRNA expression analysis.

**Table 1:** Clinical and total RNA characteristics of *SARS-CoV-2* patients and controls included in the study.

Group 1 (Covid-19 positive, persistent olfactory symptoms)											
Code	Age	Sex		Positive test date	Olfactory test date	Olfactory test score	OE sampling date	Total RNA (μg)	A260/280	A260/230	R.I.N.
P11	20	Female		2020-11-26	2021-03-29	14	2021-03-29	6.73	2.13	1.99	8.8
P12	62	Male		2020-03-27	2021-03-29	25.5	2021-03-29	6.68	2.12	1.42	8.4
P14	41	Female		2020-03-19	2021-03-29	27	2021-03-29	5.64	2.13	2.18	8.2
P16	22	Male		2020-11-01	2021-04-19	9	2021-04-19	5.13	2.11	2.22	8.6
P17	26	Female		2020-11-28	2021-04-19	29	2021-04-19	8.83	2.12	2.12	9.4
P21	48	Female		2020-03-16	2021-05-10	26	2021-05-10	2.05	2.12	2.18	7.3
Group 2 (Covid-19 positive, full recovery from olfactory symptoms)											
Code	Age		Sex	Positive test date	Olfactory test date	Olfactory test score	OE sampling date	Total RNA (μg)	A260/280	A260/230	R.I.N.
P4	28		Male	2020-10-09	2021-03-15	32.25	2021-03-15	2.15	2.19	1.32	6.4
P5	59		Female	2020-10-10	2021-03-22	35.25	2021-03-22	7.06	2.14	2.15	6.8
P18	28		Male	2020-03-04	2021-04-26	35.75	2021-04-26	6.76	2.13	1.96	8.3
P23	27		Male	2020-03-22	2021-05-24	37.75	2021-05-24	14.29	2.11	2.13	6.7
P25	59		Female	2020-03-21	2021-05-31	35.5	2021-05-31	11.82	2.11	2.2	6.5
Group 3 (Covid-19 positive, no olfactory symptoms)											
Code	Age		Sex	Positive test date	Olfactory test date	Olfactory test score	OE sampling date	Total RNA (μg)	A260/280	A260/230	R.I.N.
P6	26		Female	2020-11-11	2021-03-22	34.5	2021-03-22	6.29	2.14	2.18	6.5
P7	23		Female	2020-09-30	2021-03-22	30.5	2021-03-22	5.80	2.16	1.43	7.1
P9	37		Female	2020-11-03	2021-03-22	32.5	2021-03-22	7.04	2.14	2.05	7.3
P8	48		Male	2020-03-14	2021-03-22	35.5	2021-03-22	6.17	2.14	2.09	6.7
P22	31		Female	2020-10-30	2020-05-10	35.5	2021-05-10	2.37	2.12	2.38	7.3
Group 4 (Covid-19 negative, healthy)											
Code	Age		Sex	Positive test date	Olfactory test date	Olfactory test score	OE sampling date	Total RNA (μg)	A260/280	A260/230	R.I.N.
C5	25		Male		2021-03-29	32.5	2021-03-29	4.87	2.14	2.2	6.0
C6	27		Male		2021-03-29	30.5	2021-03-29	4.67	2.12	2.19	8.0
C9	28		Male		2021-05-10	40	2021-05-10	12.53	2.13	2.46	6.0
C10	30		Female		2021-06-14	37,5	2021-06-14	3.44	2.11	1.83	6.0
C11	27		Male		2021-06-14	43.75	2021-06-14	2.88	2.11	2.12	6.7

The olfactory test score represents functional anosmia (score 0-15), hyposmia (score 15-30), normosmia (score 30-45). R.I.N. (RNA integrity number) ranges from 1 (RNA completely degraded) to 10 (best quality RNA).

### 2.3 miRNA expression profiling

The analysis of miRNA expression profiles was performed using “Agilent SurePrint G3 human, 21^st^ version (8x60k)” microarray (Agilent Technologies, Santa Clara, CA, USA), which allows the detection of 2,549 human miRNAs (miRBase 21.0^th^ version) and 76 viral miRNAs (GEO platform N. GPL24741). Each miRNA was targeted by 16 to 20 array-probes of different sizes. Total RNA (200 ng) was labeled with pCp Cy3, according to the Agilent’s protocol, and unincorporated dyes were removed with MicroBioSpin6 columns (BioRad, Hercules, CA, USA) (29). Probes were hybridized at 55°C for 22 hours using the Agilent’s hybridization oven, which is suitable for bubble-mixing and microarray hybridization processes. Slides were examined using Agilent microarray scanner (model G2565CA) at 100% and 5% sensitivity settings. Agilent Feature Extraction software version 12.0.0.7 was used for image analysis of miRNA expression arrays. Raw miRNA data are available in the U.S National Centre for Biotechnology Information Gene Expression Omnibus (GEO, http://www.ncbi.nlm.nih.gov/geo) database with the Accession GSE209806.

### 2.4 RNA-seq

The RNA-Seq of each individual sample was carried out from IGA Technology Services (Udine, Italy). cDNA libraries were constructed with 100 ng of total RNA by using “Universal Plus™ Total RNA-Seq with NuQuant kit” (Tecan Genomics, Redwood City, CA) following the manufacturer’s instructions. The workflow consists of fragmentation of total RNA and cDNA synthesis with a mixture of random and oligo (dT) primer, followed by end repair to generate blunt end, ligation of UDI adaptors, strand selection, AnyDeplete to remove unwanted transcript, such as ribosomal RNA, and PCR amplification to generate the final library. The libraries were quantified with the Qubit 2.0 Fluorometer (Invitrogen, Carlsbad, CA, USA) and quality tested by Agilent 2100 Bioanalyzer High Sensitivity DNA Assay. Sequencing was carried out in paired-end mode (150 bp) by using NovaSeq 6000 (Illumina, San Diego, CA) with a targeted sequencing depth of about 80 million reads per sample. Raw data were processed with the software CASAVA v1.8.2 (Illumina) for both format conversion and demultiplexing. Sequence reads are available on NCBI BioProject database with the accession number PRJNA806721. Raw reads were trimmed to remove adapter sequences using cutadapt (version 3.4). The abundances of all human transcripts annotated by GENCODE (release 38) were estimated using Salmon software (version 1.5.2) (30) and then summarized at the gene level using tximport (version 1.20.0) (31). Genes were filtered genes by their expression levels using the strategy described in Chen et al. (32), as implemented in the edgeR package with default parameters, 22.612 genes were retained. Sample P21 (Group 1) was removed from the analysis due to the low amount of total RNA available.

### 2.5 Statistical analysis of miRNA and gene expression data

Inter-array normalization of miRNA expression levels was performed with cyclic Lowess for miRNA (33), the average of replicates being used. Feature Extraction software (Agilent Technologies) was employed to obtain spot quality measures for evaluating the quality and the reliability of the hybridization. In detail, the flag “glsFound” (set to 1 if the spot had an intensity value significantly different from that of the local background, 0 otherwise) was used to filter out unreliable probes: a flag equal to 0 was noted as “not available” (NA). In order to make a robust and unbiased statistical analysis, probes with a high proportion of NA values were removed from the dataset. NA (44%) was used as threshold in the filtering process, a total of 210 available human miRNAs being obtained. Differentially expressed miRNAs were identified with two class-Significance Analysis of Microarray (SAM) algorithm (34) with default settings. SAM, which uses a permutation-based multiple testing algorithm, associates a variable false discovery rate (FDR) with the significant genes. FDR, which refers to the percentage of error that can occur in the identification of the statistically significant differentially expressed miRNAs in multiple comparisons, can be manually adjusted (FDR < 0.05). Samples P16 (Group 1), P6 (Group 3), and C9 (Group 4) were excluded from the analysis due to issues in the miRNA labelling step; sample P16 was replaced with sample P12, belonging to the same Group but not selected for gene expression analysis.

Gene-level counts deriving from RNA-Seq were normalized using RUVseq (version 1.26.0; RUVg method, k=7 confounding factors) (35). Differential expression was tested with edgeR (version 3.34.1) (36), using the GLM model. Genes with an adjusted p-value (FDR) < 0.10 after correction for multiple testing (Benjiamini-Hochberg method) were considered differentially expressed. Finally, to analyze the functional relationship of these differentially expressed genes, a Gene Ontology (GO) functional enrichment analysis through the ShinyGO tool (FDR < 0.05) was performed (37). Sample C10 (Group 4) was excluded from the analysis due to his low sequencing depth.

All the heat maps were obtained by Morpheus software (https://software.broadinstitute.org/morpheus, Broad Institute, USA) using an unsupervised two-dimensional hierarchical clustering approach with the average linkage method and Euclidean correlation.

### 2.6 Reverse transcription of RNA and quantitative PCR

First-strand cDNA synthesis was performed with “GoScript™ Reverse Transcriptase kit” (Promega, Madison, WI, USA) starting from 500 ng of total RNA in a final volume of 20 μL according to the manufacturer’s instructions. qRT-PCRs were performed in triplicate using Bio-Rad CFX 384 Touch System (Bio-Rad, Hercules, CA, USA) and “GoTaq^®^ qPCR Master Mix” chemistry (Promega, Madison, WI, USA). The qPCR cycling conditions were 95°C for 2 min, 39 cycles (95°C for 25 s and 60°C for 1 min), and a final step at 72°C for 3 min. The 2^-ΔΔCt^ (RQ, relative quantification) and ΔCt (Ct_(GOI)_ – Ct_(end, ctl)_) method were used to calculate the relative expression ratio and individual expression level, respectively. The oligonucleotides employed are shown in **Supplementary Table 1**, *Beta-2-microglobulin* (*B2M*) was used as endogenous control. Samples P4 (Group 2) and P22 (Group 3) were not included because of insufficient remaining amount of total RNA.

For the quantification of miRNA expression levels, cDNA was synthesized using the miRCURY LNA RT Kit (Qiagen, Hilden, Germany) starting from 10 ng of total RNA with the addition of 0.5 μL of *UniSp6* as exogenous miRNA spiked-in control. PCR was performed in a 10 μL volume containing 5 μL 2x miRCURY SYBR Green Master Mix (Qiagen, Hilden, Germany), 3 μL cDNA of a dilution 1:60, 1 μL RNase-free water, and 1 μL of one of the following miRCURY LNA PCR primer sets (Qiagen, Hilden, Germany): *hsa-miR-16-5p* (ID YP00205702), *hsa-miR29a-3p* (ID YP00204698), *hsa-miR-21-5p* (ID YP00204230) and *UniSp6* (ID ZP00004674). The qPCR reactions were performed in a Bio-Rad CFX 96 Touch System (BioRad, Hercules, CA, USA). The qPCR cycling conditions were 95°C for 2 min and 40 cycles (95°C for 10 s and 56°C for 1 min). Three replicates of each sample were amplified for each real-time PCR reaction. The relative expression levels between samples were calculated using the comparative delta Ct (threshold cycle number) method (2^-ΔΔCt^). *hsa-miR-16* and C6 patient were used as endogenous control and as calibrator sample respectively.

## 3 Results

### 3.1 Patient enrolment

For this study, 21 subjects were enrolled after at least 4 months from the infection. All the subjects were tested for smell sensitivity by adopting the Sniffin’ sticks and sorted into four Groups according to their olfactory symptoms (**Supplementary Figure 1**). Group 1 was composed by 6 patients recovered from COVID-19 infection by at least 3 months but still manifesting olfactory symptoms. Group 2 was composed by 5 patients who experienced olfactory symptoms during the infection, that resolved 3-4 weeks after recovery. Group 3 was composed by 5 patients who did not experience olfactory symptoms during and after the infection. Group 4 was composed of 5 healthy subjects who were never been diagnosed with *SARS-CoV-2* infection and never complained of smell loss. Clinical details of the enrolled subjects relevant for this study are reported in **Table 1**.

### 3.2 Samples are enriched in olfactory epithelial cells

**Figure 1:**
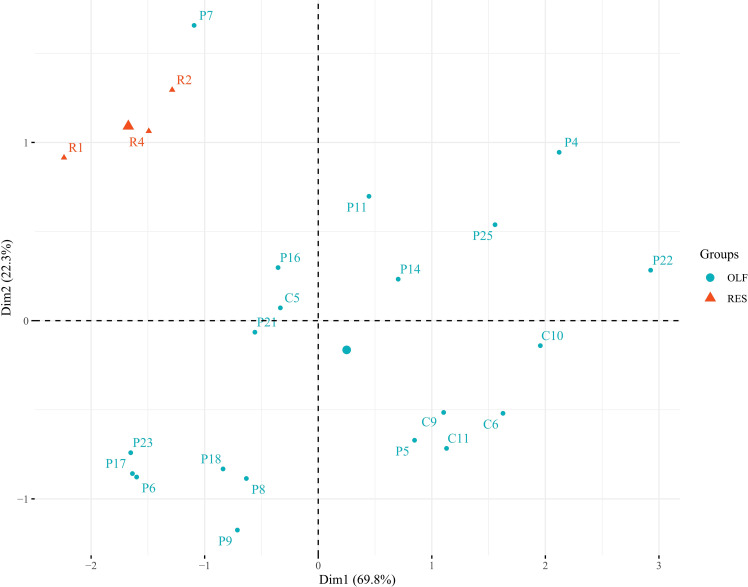
Principal component analysis (PCA) on olfactory epithelium cell markers (*krt5*, *cyp2j2* and *cyp2a13*) tested in olfactory (light blue) and respiratory (orange) epithelium samples. Two distinct clusters (with the only exception of P7 sample) were obtained demonstrating a clear enrichment for the olfactory epithelium cells in the samples selected and used for the subsequent analysis.

In order to check the enrichment in olfactory epithelium cells, the expression levels of *Keratin 5* (*krt5*), marker of horizontal basal cells, and cytochromes *cyp2j2* and *cyp2a13*, markers of sustentacular cells, were quantified and compared to those of respiratory epithelium samples; the ΔCts were used for a principal component analysis (PCA) (38–40). As shown in **Figure 1**, PCA discriminates all the olfactory and respiratory epithelium samples in two different Groups, demonstrating a clear enrichment in olfactory epithelium cells in the samples of interest, with the only exception of P7 sample who has been removed from the following analysis due to its poor enrichment in olfactory epithelial cells.

### 3.3 miRNOME remains altered after several months from *SARS-CoV-2* infection

**Figure 2:**
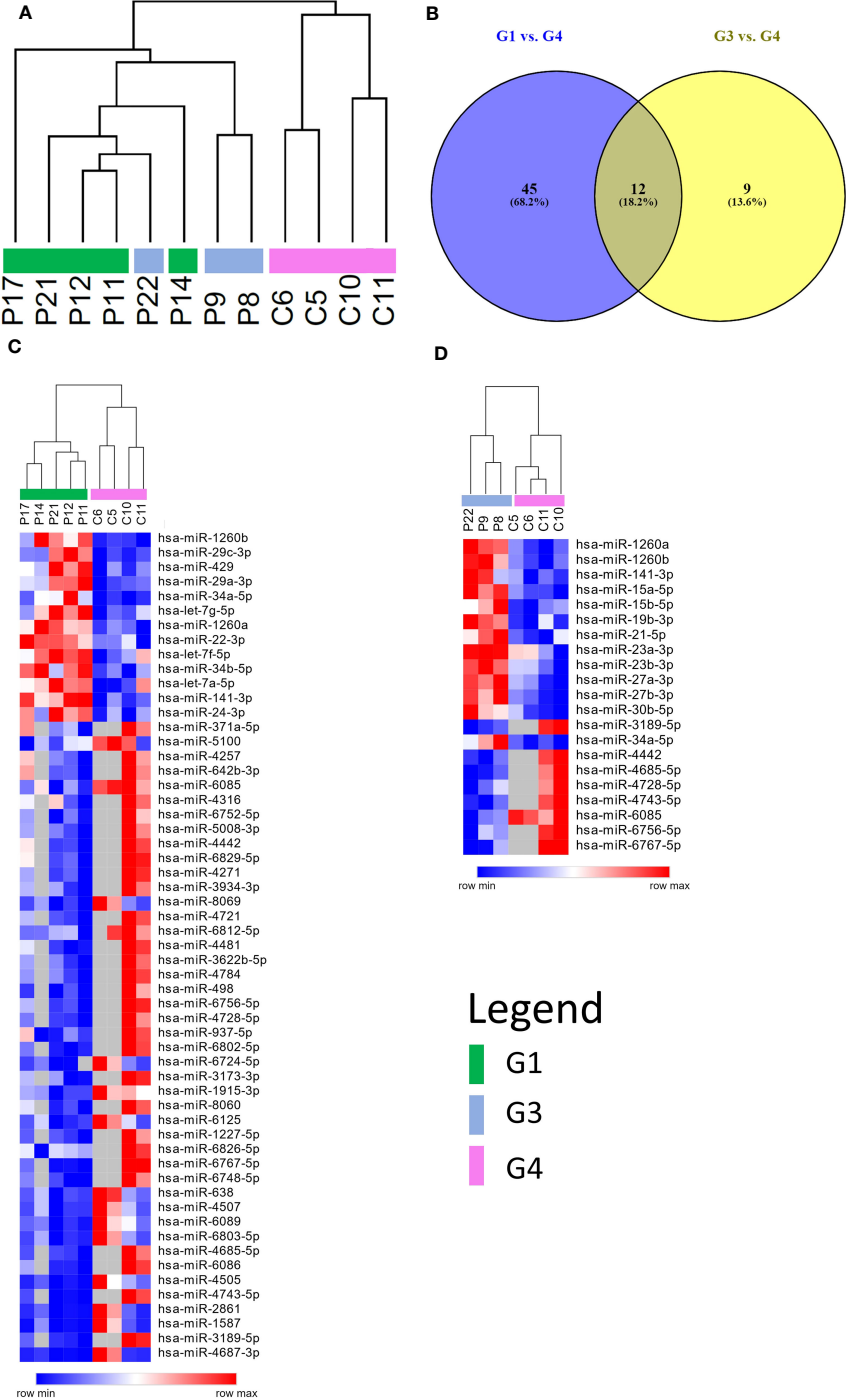
miRNAs expression profiling. **(A)** Unsupervised hierarchical clustering analysis by using the 210 detected miRNAs. Patients are clearly separated from healthy controls, but not according to the severity of the olfactory symptoms. **(B)** Venn diagram of differentially expressed miRNAs resulting from the comparisons between persistent and no olfactory symptoms patients with respect to healthy controls. Most of differentially expressed miRNA are the same in both comparisons. Heatmap representing differentially expressed miRNAs of two different comparisons: persistent olfactory symptoms **(C)** and no olfactory symptoms **(D)** patients with respect to healthy controls. Most of miRNAs are under expressed after *SARS-CoV-2* infection. A complete list of differentially expressed miRNAs is provided in the **Supplementary Materials** (**Supplementary Tables 2**, **3**).

**Figure 3:**
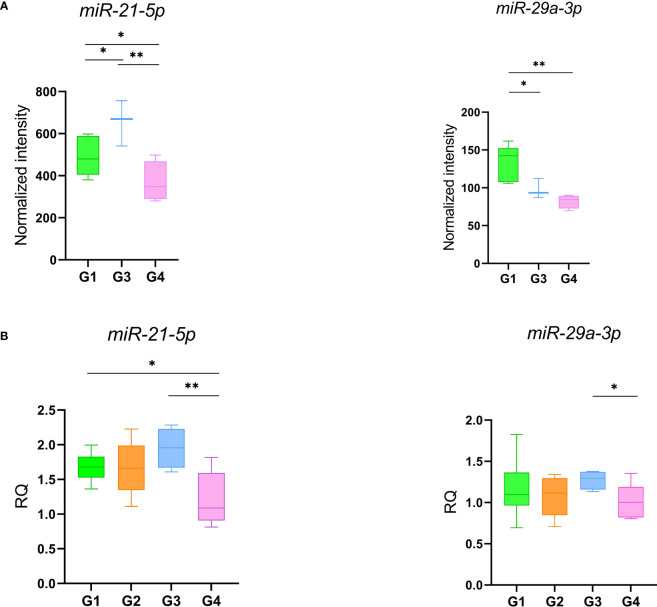
*miR-21* and *miR-29* expression levels obtained with microarray **(A)** and qRT-PCR **(B)**. Data were compared by t-test *p-value ≤ 0.05 **p-value ≤ 0.01.

A microarray analysis was performed to identify specific microRNA (miRNA) expression signatures in patients with persistent olfactory symptoms (Group 1) and patient who never had olfactory symptoms (Group 3), using healthy subjects (Group 4) as controls. An unsupervised hierarchical clustering analysis, by using the expression levels of 210 detected miRNAs, was able to clearly separate *SARS-CoV-2* patients from healthy controls, but not the patients with different olfactory symptoms (**Figure 2A**). 57 differentially expressed miRNAs were found between Group 1 and healthy controls (Group 4) (**Supplementary Table 2**), 21 miRNAs were differentially expressed between Group 3 and Group 4 (12 of which were also found in the comparison between Groups 1 and 4, **Figure 2B**) (**Supplementary Table 3**) and 4 miRNAs were differentially expressed between Group 1 and Group 3 with FDR < 0.05 (**Supplementary Table 4**). The large majority of differentially expressed miRNAs in these comparisons were under-expressed in patients with respect to healthy controls, even months after negative PCR tests, suggesting that the expression levels of these miRNAs is affected by *SARS-CoV-2* infection well beyond its acute phase (**Figures 2C, D**). Among the overexpressed miRNAs, we identified miRNAs involved in neuronal development (let-7 family, including *let-7a-5p*, *let-7f-5p*, *and let-7g-5p*, **Supplementary Figure 2**) (41) and miRNAs with pro-inflammatory activity (miR-34 family, including *miR-34a-5p* and *miR-34b-5p*, **Supplementary Figure 2**) (42). In addition, *miR-21-5p* and *miR-29a-3p*, that take part in immune cells differentiation, were also overexpressed in patients with respect to healthy controls (**Figure 3A**). The expression levels of *miR-21-5p* and *miR-29a-3p* were also confirmed by using qRT-PCR (**Figure 3B**).

### 3.4 Gene expression profiling reveals a dysregulation of immune response and zinc homeostasis

**Figure 4:**
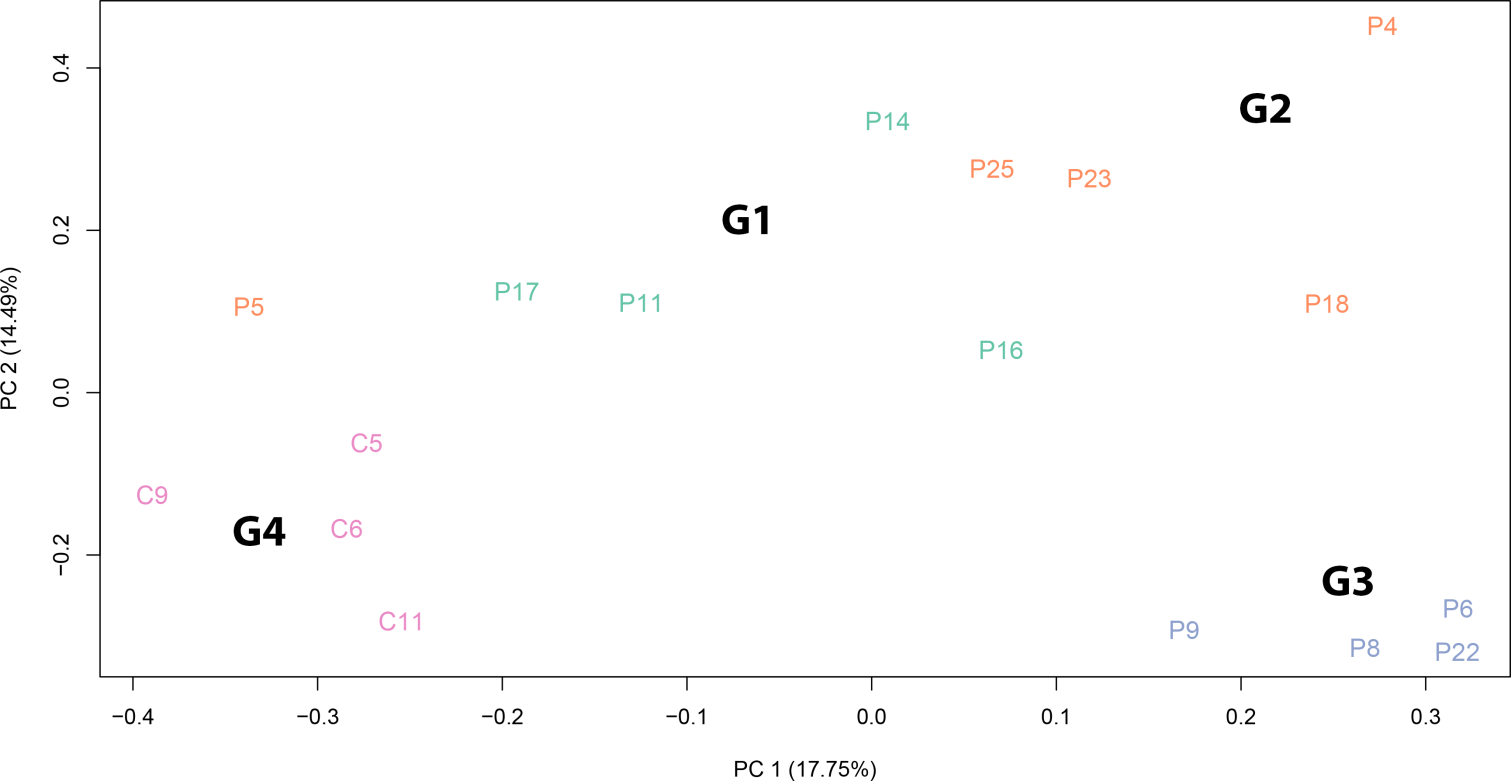
Principal component analysis of normalized RNA-Seq read counts. The PCA shows the variance-stabilized gene expression dataset containing 13 *SARS-CoV-2* patients and 4 healthy controls. Different colours indicate samples belonging to different groups: patients with persistent olfactory symptoms (Group 1, green), patients with full recovery of olfactory perception (Group 2, orange), patient with no olfactory symptoms (Group 3, light blue) and healthy controls (Group 4, pink). The PCA was able to separate samples according to *SARS-CoV-2* infection and to different olfactory symptoms. The explained variance (%) for PC1 and PC2 are also given.

To assess whether the *SARS-CoV-2* infection was accompanied by differential expression of specific set of genes, we performed an RNA-Seq analysis of total RNA extracted from 18 subjects (**Table 1**). A principal component analysis (PCA) clearly revealed that patients belonging to different Groups are characterized by specific gene expression signatures. The PCA was able to clearly stratify patients by attributing them to the Groups in which they were classified from a clinical point of view. Gene expression signatures clearly separate *SARS-CoV-2* patients from healthy controls and also patients with different olfactory symptoms (**Figure 4**). Interestingly, patient P5 (Group 2) is localized closer to the patients of Group 1 and this could be due to the fact that this patient at the moment of olfactory cell sampling reported to the clinicians to have yet some olfactory symptoms even if its TDI (35.25) indicated normosmia. We decided to exclude this sample from the subsequent analysis because it might have been improperly included in the Group 2. Paired comparisons revealed 206 (G1 vs. G2, 118 up and 88 down), 637 (G1 vs. G3, 459 up and 178 down), and 307 (G1 vs. G4, 195 up and 112 down) (LFCT=1, BH adjusted p-value < 0.05) differentially expressed genes (DEGs). Further, 412 (316 up and 96 down) and 810 (379 up and 431 down) DEGs were found between patients with a full recovery of olfactory perception (Group 2) and those with no olfactory symptoms (Group 3) and the healthy ones (Group 4) respectively. Finally, 676 DEGs (147 up and 529 down) were found between healthy controls and patients with no olfactory symptoms (Group 3) (**Supplementary Table 5**). Interestingly, the large majority of differentially expressed genes were downregulated in *SARS-CoV-2* patients (Group 2 and 3) with respect to healthy controls. On the contrary, we observed a clear up-regulation of DEGs in patients with persistent olfactory symptoms (Group 1) with respect to patients with transient olfactory symptoms (Group 2), the ones with no olfactory symptoms (Group 3) and healthy controls (Group 4).

**Figure 5:**
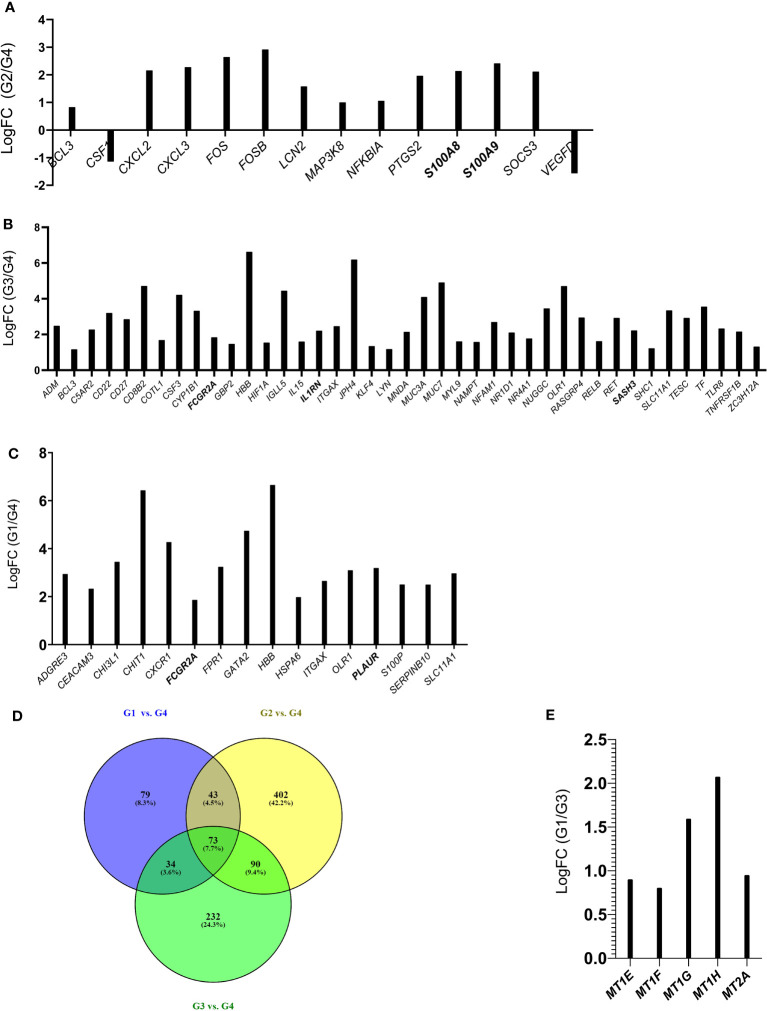
Histograms of DEGs belonging to the IL-17 and TNF pathway (Group 2 vs. Group 4) **(A)**; Biological Processes related to immune activities (Group 3 vs. Group 4) **(B)** and to neutrophils and granulocytes (Group 1 vs. Group 4) **(C)**. Common DEGs between G1 vs. G4, G2 vs. G4, G3 vs. G4 comparisons are represented in the Venn Diagram **(D)**, Histograms of DEGs belonging to the Biological Process of detoxification of inorganic compound (Group 1 vs. Group 3) **(E)**. In bold are indicated the genes that were validated by qRT-PCR (**Supplementary Figure 7**).

Both the comparisons between patients without olfactory symptoms at the time of the cells sampling (Group 2 and Group 3) *versus* healthy controls (Group 4) are characterized by DEGs that showed an enrichment of biological processes involved in vasculature and endothelium development as well as in immune activities (**Figure 5A**) known to be triggered by bacterial/Lipopolysaccharide (LPS) stimuli (**Supplementary Figures 3, 4**, **Supplementary Tables 6–8**). In addition, the comparison between patient recovered from the olfactory symptoms and healthy controls (Group 2 *vs.* Group 4) also highlighted an enrichment in interleukin-17 (IL-17) and tumor necrosis factor (TNF) pathways, suggesting an immune response characterized by Th17 cells in the olfactory epithelium (**Figure 5B**, **Supplementary Figure 5**, **Supplementary Table 9**) of those individuals. Finally, the comparisons between patients with persistent olfactory symptoms (Group 1) and healthy controls (Group 4) showed an enrichment in biological processes involved in neutrophils, myeloid and granulocytes activities (**Figure 5C**, **Supplementary Figure 6**, **Supplementary Table 10**). Nine differentially expressed genes obtained from these comparisons were successfully validated by qRT-PCR, confirming the RNA-Seq data (**Supplementary Figure 7**).

Interestingly, the lists of differentially expressed genes obtained in the following comparisons G1 vs. G4, G2 vs. G4 and G3 vs. G4 highlighted common DEGs (**Figure 5D**) that could be related to infection with *SARS-CoV-2.* Only two genes (*ITGAX* and *FCGR2A*) of the list of common DEGs (73 genes, and **Supplementary Table 11**) were found among G1 vs. G4 belonging to “neutrophil and granulocyte activation” biological process. If we consider the common DEGs between the G1 vs. G4 and G3 vs. G4 comparisons (107 genes, **Supplementary Table 11**), in addition to *ITGAX* and *FCGR2A* genes we find three other genes (*HBB*, *OLR1*, *SLC11A1*) that are involved in the inflammatory process. Overall, this analysis highlights an inflammatory/immune response triggered by *SARS-CoV-2* infection. While this finding was not unexpected, it is surprising that this molecular signature is still present after months from COVID-19 diagnosis.

**Figure 6:**
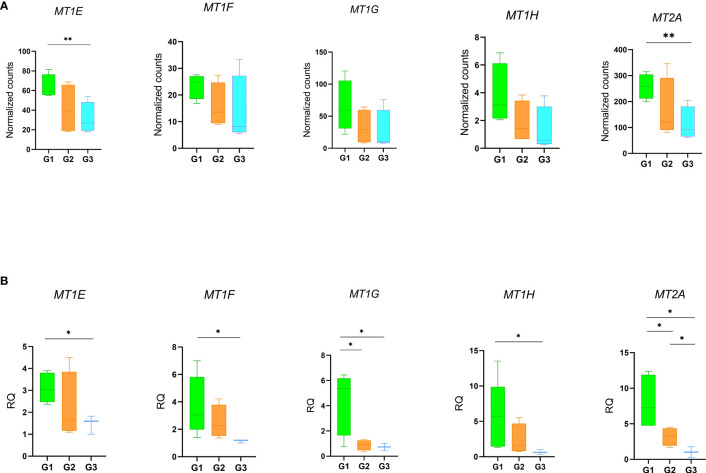
Gene expression profiles of metallothioneins. Expression levels of the MTs in patients with persistent olfactory symptoms (Group 1), with full recovery of olfactory perception (Group 2) or who never experienced olfactory symptoms (Group 3) was measured by RNA-Seq **(A)** and qRT-PCR **(B)**. Data were compared by t-test *p-value ≤ 0.05, **p-value ≤ 0.01.

Notably, the GO analysis performed on DEGs between patients with persistent olfactory symptoms (Group 1) and patients who never experienced these symptoms (Group 3) after the infection revealed a statistically significant enrichment of genes involved in detoxification of inorganic compound (*MT1E*, *MT1F*, *MT1G*, *MT1H* and *MT2A*, **Figure 5E**, **Supplementary Figure 8**, **Supplementary Table 12**). These genes encode for different metallothioneins, small proteins which bind and sequester zinc to reduce its toxic effects in the cell. Accordingly, their expression levels were shown to rapidly increase after zinc exposure or inflammatory processes (43). Interestingly, we observed a relationship between expression of metallothioneins and the olfactory symptoms reported by patients of different Groups. The level of expression of metallothioneins, obtained by RNA-Seq (**Figure 6A**) and qRT-PCR (**Figure 6B**), were the lowest in patients who never experienced olfactory disorders, intermediate in patients fully recovered from olfactory disorders, and the highest in patient with persistent olfactory symptoms.

## 4 Discussion

**Figure 7:**
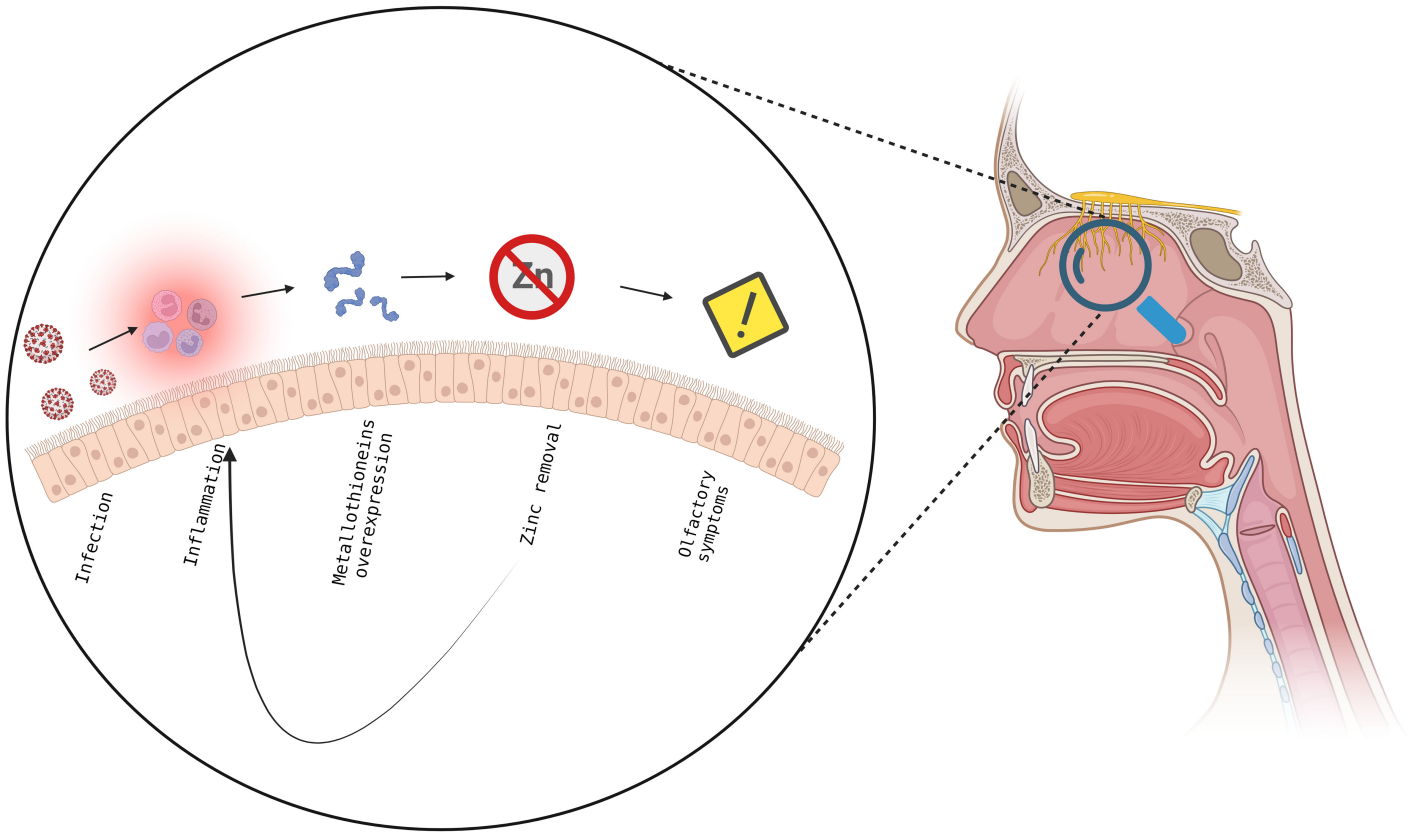
Proposed model to explain the insurgence and maintenance of olfactory symptoms in *SARS-CoV-2* pathology. In the olfactory epithelium, the viral infection leads to an inflammatory state and overexpression of metallothioneins, with subsequent decrease in zinc levels, resulting in immune response and in the insurgence of olfactory symptoms establishing a feedback mechanism which does not allow the full recovery from olfactory symptoms. Created with BioRender.com.

The aim of this study was to identify miRNA and gene expression profiling from the cells of olfactory epithelium of patients with persistent olfactory symptoms (Group 1), recovered from olfactory perturbation (Group 2) and subjects who never experienced these symptoms (Group 3) after the infection of *SARS-CoV-2*, in order to highlight the mechanism underlying the different clinical manifestation. For this purpose, 21 subjects were recruited, and the olfactory epithelium were sampled, RNA extracted and analysed. All samples show an enrichment of olfactory epithelial cells, with the only exception of the P7 sample which has been removed from the study. The miRNome resulted significantly altered after several months from *SARS-CoV-2* infection, with an alteration in expression levels of miRNAs involved in neuronal development (ie., let7 family) and immune response, such members of the miR-21 and miR-29 families. Interestingly, *miR-21-5p* overexpression leads to the inhibition of T helper 1 (Th1) immune response (44). Further, miR-21 and miR-29 families overexpression leads to an inhibition of Th1 and Th17 differentiation (45, 46) and they could affect the immune response, as Th1 responses are the most effective against viruses (47). It is possible that, regardless of severity of symptoms, miRNA can affect both neuronal development and immune response. However, our analysis did not detect a significant association of miRNA with the severity of the olfactory symptoms. RNA-Seq analysis show for all patients of Groups 1-3 a prolonged immune response compared to healthy subjects (Group 4). Interestingly, *FCGR2A* and *ITGAX* (also known as *CD11c*) both surface marker of neutrophils, resulted upregulated in all the comparisons between patients and healthy controls (48, 49). These findings support an expected general inflammatory/immune response towards *SARS-CoV-2* infection. What is surprising is the fact that this molecular signature persists after 4 or more months after COVID-19 diagnosis. Consistent with our results, a recent proteomic analysis, conducted on peripheral blood mononuclear cell (PBMCs) isolated from healthy controls and patients with modest or severe COVID-19 disease, showed an upregulation of proteins involved in neutrophils activation and degranulation in patients with severe disease, suggesting a role of neutrophils in local or systemic COVID-19 pathogenesis. It has been shown that the viral non-structural protein 10 (nps10) interacts with nuclear factor-kB-repressing factor (NKRF) favouring the expression of IL-8 and the recruiting of neutrophils (50). Change in the expression of genes observed in the comparison of patients with persistent olfactory symptoms (Group 1) and subjects who never experienced these symptoms (Group 3) are consistent with a dysregulation of the response to metal and zinc ions. Since patients were not taking zinc dietary supplements, it is reasonable to hypothesize that the over-expression of metallothioneins (MT) might be due to the inflammatory state occurring at the level of the olfactory epithelium in these patients, given their regulatory role in immunity (51). In particular, in the CNS, several studies have pointed at a neuroprotective role of MTs against brain injuries. It has been shown that MT are produced in response to inflammatory cytokines, such as IL-6, which is a cytokine induced by *SARS-CoV-2* infection and is critically associated with disease severity (52, 53). Overexpression of MTs results in decreased zinc levels. Zinc is an essential micronutrient that regulates the immune system functions, and its low nasal levels represent a local immune response to acute viral infections, *SARS-CoV-2* included. Importantly, zinc deficiency is known to induce loss of smell and taste (54–56). Although the role of zinc in neurons physiology is still debated, it may act as a second messenger, taking part in neuronal receptor signaling (57). From an immunological viewpoint, MTs are known to be produced in states of inflammation, in response to cytokines (including Il-6) and, by regulating redox status, they can protect the host from some of the toxic effect of ROS, which are produced by neutrophils and are known to be implicated in the immunopathogenesis of COVID-19. MT expression is associated with differentiation of Treg cells, while inhibiting Th17 and Th1 differentiation (51). Localizing on the cell surface, MTs sequestering zinc ion can interfere with CD-T cell interaction, stalling the TCR signaling (51). A reduction in zinc level can also affect the killing ability of cytotoxic T lymphocytes (CTLs) (51, 58–60). Furthermore, an alteration of the zinc level can promote a dendritic cell maturation and antimicrobial response of neutrophils and macrophages (51). It has been proposed that a drop in the local zinc level caused by *SARS-CoV-2* may decrease type 1 interferons (60), which is critically involved in the control of *SARS-CoV-2* replication (61–65). Interestingly, it has been hypothesized that zinc deficiency could be a predisposing factor for *SARS-CoV-2* infection (66). Furthermore, studies have associated serum zinc levels at the onset with the severity of COVID-19 symptomatology (67–70) and with response to vaccine (71). In case of persistence, low amounts of zinc could result in enhanced replication with systemic viral spread and more severe symptoms (60). Clinical trials are ongoing to evaluate the effect of zinc supplementation and association with antiviral compounds in COVID-19 patients (69, 72). In conclusion, our data suggest that patients with persistent olfactory symptoms present an abnormal inflammation of the olfactory epithelium, resulting in high levels of active MT, which sequestering zinc enhance the local inflammation, creating a feed-back loop and preventing a full recovery of these patients (**Figure 7**) (73, 74). Furthermore, several proteins involved in epigenetic regulation (e.g. DNA methyltransferases, histone acetylases, and transcription factors with a zinc-binding domain), require zinc to work properly, and the role of this ion in this process has already been demonstrated (75, 76). We speculate that zinc removal caused by MT over-expression compromises the activity of proteins involved in epigenetic regulation, promoting DNA reorganization and the related down-regulation of genes involved in odor sensing. Because of their effects both on neurological and immunological mechanisms of pathogenesis, MT may be attractive targets to mitigate symptoms of COVID-19.

## Data availability statement

The datasets presented in this study can be found in online repositories. The names of the repository/repositories and accession number(s) can be found here: GSE209806 (GEO) and PRJNA806721 (BioProject).

## Ethics statement

The studies involving human participants were reviewed and approved by Ethical Committee, Azienda Ospedaliera di Padova, Padova, Italy. The patients/participants provided their written informed consent to participate in this study.

## Author contributions

LL: investigation, methodology, data analysis and curation, validation, writing—original draft, writing—review and editing. AB: investigation, clinical data. GS: statistical analysis and curation. DC: methodology, data analysis, writing—review and editing. AV: investigation; methodology. ABi: validation, data analysis and curation. AR: investigation, methodology. AG: supervision, writing—review and editing. AA: funding acquisition, conceptualization. GO: investigation, clinical data, writing—review and editing. CM: investigation, methodology, writing—review and editing. CP: funding acquisition, conceptualization, writing—review and editing. AC: funding acquisition, conceptualization, supervision, writing—original draft, writing—review and editing. CDP: conceptualization, supervision, writing—original draft, writing—review and editing. All authors contributed to the article and approved the submitted version.
