# Supplementary material for: Persistent and transient olfactory deficits in COVID-19 are associated to inflammation and zinc homeostasis
Source: Front Immunol. 2023 Jul 14;14:1148595. doi: 10.3389/fimmu.2023.1148595 (PMC10380959; doi:10.3389/fimmu.2023.1148595)
Supplement: Supplementary file 1 [file DataSheet_1.docx]

Supplementary Material

Persistent and transient olfactory deficits in COVID-19 are associated to inflammation and zinc homeostasis

Lorenzo Lupi^1, 2^, Anna Bordin^3^, Gabriele Sales^1^, Davide Colaianni^1^, Adriana Vitiello^2^, Alberto Biscontin^1^, Alberto Reale^2^, Alfredo Garzino-Demo^2, 4^, Angelo Antonini^5, 6^, Giancarlo Ottaviano^3^, Carla Mucignat^2^, Cristina Parolin^2^, Arianna Calistri^2,^ *, Cristiano De Pittà^1,^ *

^1^Department of Biology, University of Padova, Padova, Italy.

^2^Department of Molecular Medicine, University of Padova, Padova, Italy.

^3^Department of Neurosciences, Otolaryngology Section, University of Padova, Padova, Italy.

^4^Department of Microbiology and Immunology, School of Medicine, University of Maryland, Baltimore, Maryland, USA.

^5^Parkinson and Movement Disorders Unit, Study Center for Neurodegeneration (CESNE), Department of Neurosciences, University of Padova, Padova, Italy.

^6^Department of Neurosciences, University of Padova, Padova, Italy.

***Correspondance:**

Cristiano De Pittà

cristiano.depitta@unipd.it

Arianna Calistri

arianna.calistri@unipd.it

# Supplementary Figures and Tables

## Supplementary Figures

Supplemental Figure 1. Schematic representation of the research workflow. Created with BioRender.com.

Supplemental Figure 2. Expression levels of some differentially expressed miRNA (*let-7a-5p*, *let-7f-5p*, *let-7g-5p,* *miR-34a-5p* and miR-34b-5p) from the comparison between patients with persistent (Group 1) and who never experienced (Group 3) olfactory symptoms with respect to healthy controls (Group 4). Data were compared by t-test. * pvalue < 0.05 ** pvalue < 0.01.

Supplemental Figure 3. Differentially expressed genes between patients with fully recovered olfactory symptoms (Group 2) and healthy controls (Group 4) were used for GO analysis. GO terms are ordered according to their FDR. The area of circle for each functional category is directly proportional to the levels of enrichment (numbers of DEGs with respect to the total of genes belonging to each category).

Supplemental Figure 4. Differentially expressed genes (A) and only the up-regulated DEGs (B) between patients who never experienced olfactory symptoms (Group 3) and healthy controls (Group 4) were used for GO analysis. GO terms are ordered according to their FDR. The area of circle for each functional category is directly proportional to the levels of enrichment (number of DEGs with respect to the total of genes belonging to each category).

Supplemental Figure 5. KEGG representation of IL-17 (A) and TNF (B) pathways significantly enriched by the GO analysis with the differentially expressed genes from the comparison between patient with a full recovery of olfactory symptoms (Group 2) and healthy controls (Group 4). DEGs are highlighted in red.

Supplemental Figure 6. Differentially expressed genes between patients with persistent olfactory symptoms (Group 1) and healthy controls (Group 4) were used for GO analysis. GO terms are ordered according to their FDR. The area of circle for each functional category is directly proportional to the levels of enrichment (numbers of DEGs with respect to the total of genes belonging to each category).

**Supplemental Figure 7.** Validation of RNA-Seq expression values by qRT-PCR.

Gene expression levels obtained by RNA-Seq and qRT-PCR are represented by histograms as LogFC (average expression value in patients of the same group compared to those of another) as indicated in Y axis. *Beta-2-microglobulin* (*B2M*) was used as endogenous control in qRT-PCR. The RNA-Seq and qRT-PCR expression profiles of each gene are shown as black and grey histograms respectively. Pearson correlation was calculated to estimate the association between RNA-Seq data and qRT-PCR results (r > 0.6 is considered as statistically significant).

**Supplemental Figure 8.** Differentially expressed genes between patients with persistent olfactory symptoms (Group 1) and patients who never experienced olfactory symptoms (Group 3) were used for GO analysis. GO terms are ordered according to their FDR. The area of circle for each functional category is directly proportional to the levels of enrichment (numbers of DEGs with respect to the total of genes belonging to each category).

## Supplementary table

**Supplemental Table 1.** List of primers employed for qRT-PCR

**Supplemental Table 2.** List of differentially expressed miRNAs (57) between patients with persistent olfactory symptoms (Group 1) and healthy controls (Group 4).

**Supplemental Table 3.** List of differentially expressed miRNAs (21) between patients who never experienced olfactory symptoms (Group 3) and healthy controls (Group 4).

**Supplemental Table 4.** List of differentially expressed miRNAs (4) between patients with persistent olfactory symptoms (Group 1) and who never experienced olfactory symptoms (Group 3).

**Supplemental Table 5**. List of differentially expressed genes (DEGs) identified from all paired comparisons between all Groups (G1 *vs*. G2, G1 *vs*. G3, G1 *vs*. G4, G2 *vs*. G3, G2 *vs*. G4, G3 *vs*. G4).

**Supplemental Table 6.** List of biological processes which are significantly enriched in the comparison between patients with transient olfactory symptoms (Group 2) and healthy controls (Group 4).

**Supplemental Table 7.** List of biological processes which are significantly enriched in the comparison between patients who never experienced olfactory symptoms (Group 3) and healthy controls (Group 4).

**Supplemental Table 8.** List of biological processes which are significantly enriched by using only upregulated differentially expressed genes obtained in the comparisons between patients who never experienced olfactory symptoms (Group 3) and healthy controls (group 4).

**Supplemental Table 9.** List of KEGG pathways which are significantly enriched in the comparison between patients with transient olfactory symptoms (Group 2) and healthy controls (Group 4).

**Supplemental Table 10.** List of biological processes which are significantly enriched in the comparison between patients with persistent olfactory symptoms (Group 1) and healthy controls (Group 4).

**Supplemental Table 11**. . List of common DEGs between the comparison G1 *vs*. G4 and G3 *vs*. G4, G1 *vs*. G4, G2 *vs*. G4 and G3 *vs*. G4 and the common DEGs between the comparison G2 *vs*. G4 and G3 *vs*. G4.

**Supplemental Table 12.** List of biological processes which are significantly enriched by differentially expressed genes between patients with persistent olfactory symptoms (Group 1) and patients who never experienced these symptoms (Group 3).
