# Supplementary figures and images for: Persistent and transient olfactory deficits in COVID-19 are associated to inflammation and zinc homeostasis
Source: Front Immunol. 2023 Jul 14;14:1148595. doi: 10.3389/fimmu.2023.1148595 (PMC10380959; doi:10.3389/fimmu.2023.1148595)

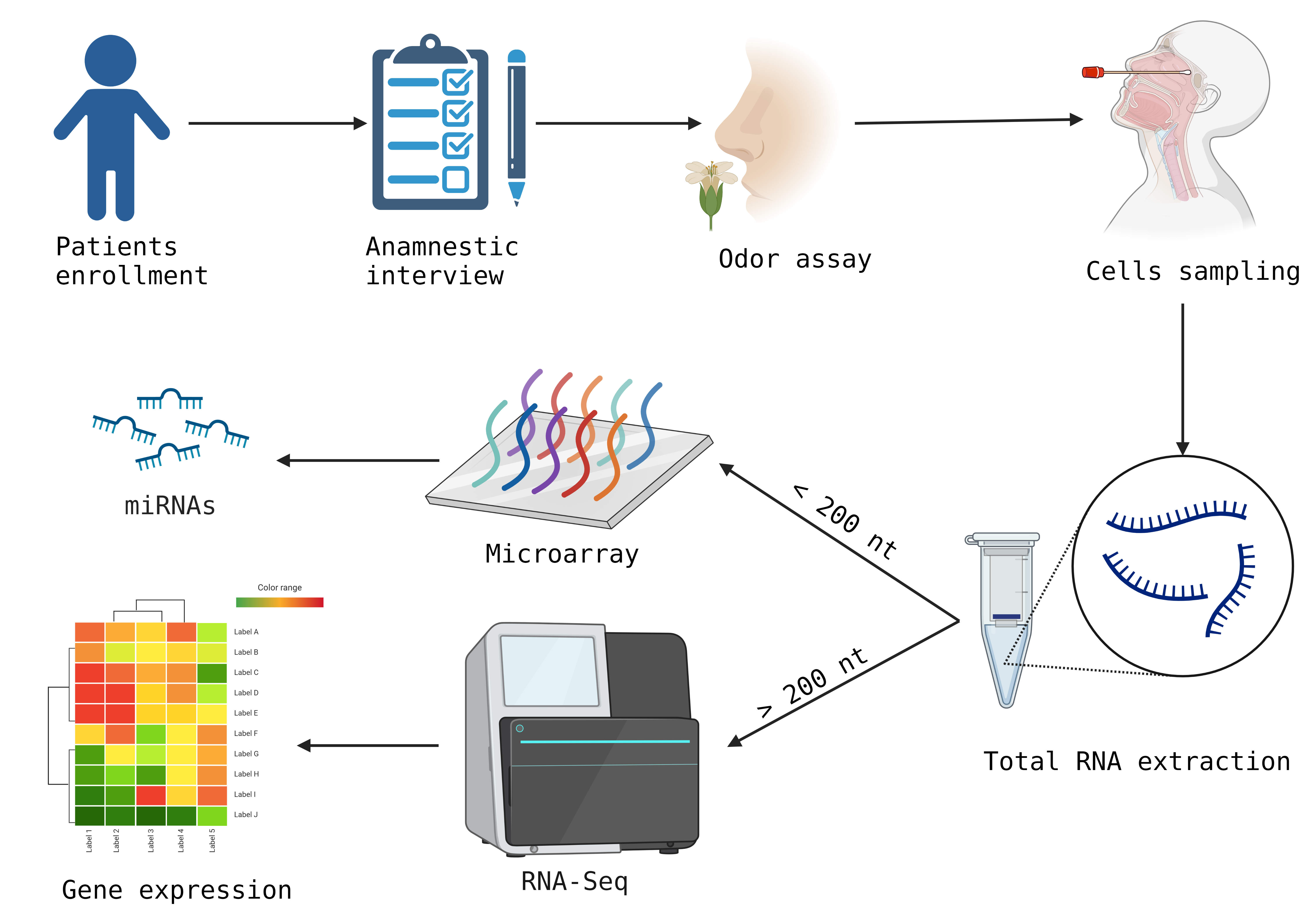

Supplement: Supplementary file 3 [file Image_1.jpeg]

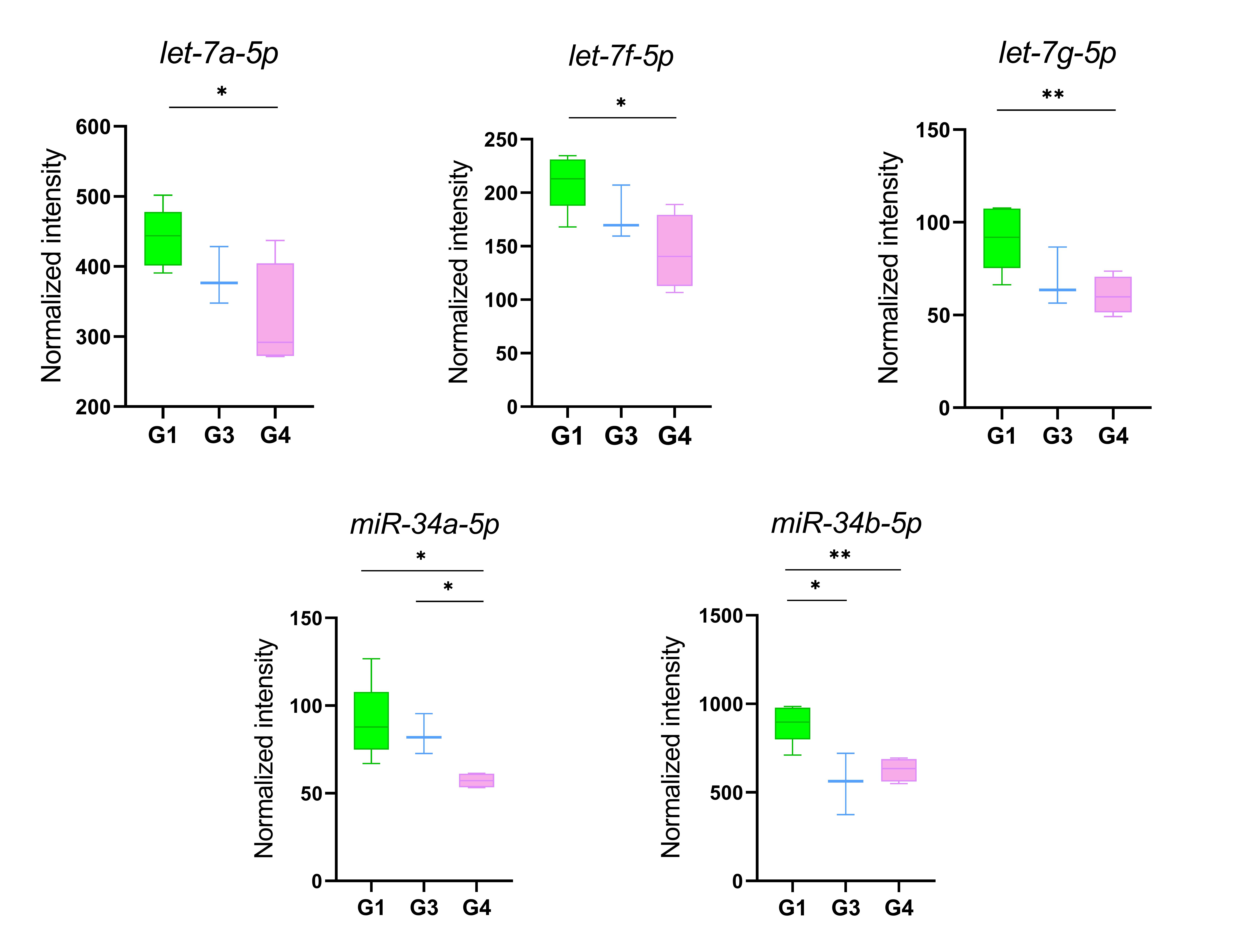

Supplement: Supplementary file 4 [file Image_2.jpeg]

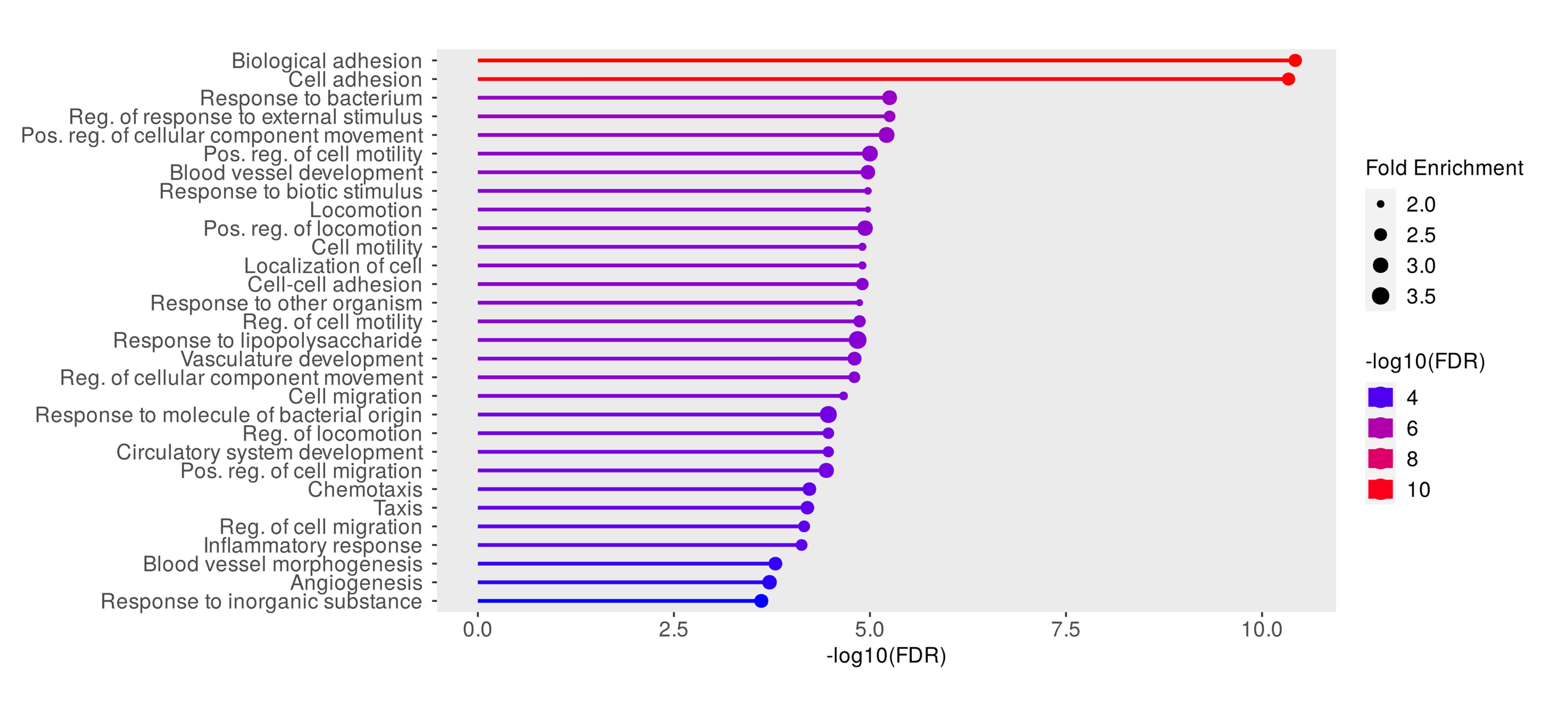

Supplement: Supplementary file 5 [file Image_3.jpeg]

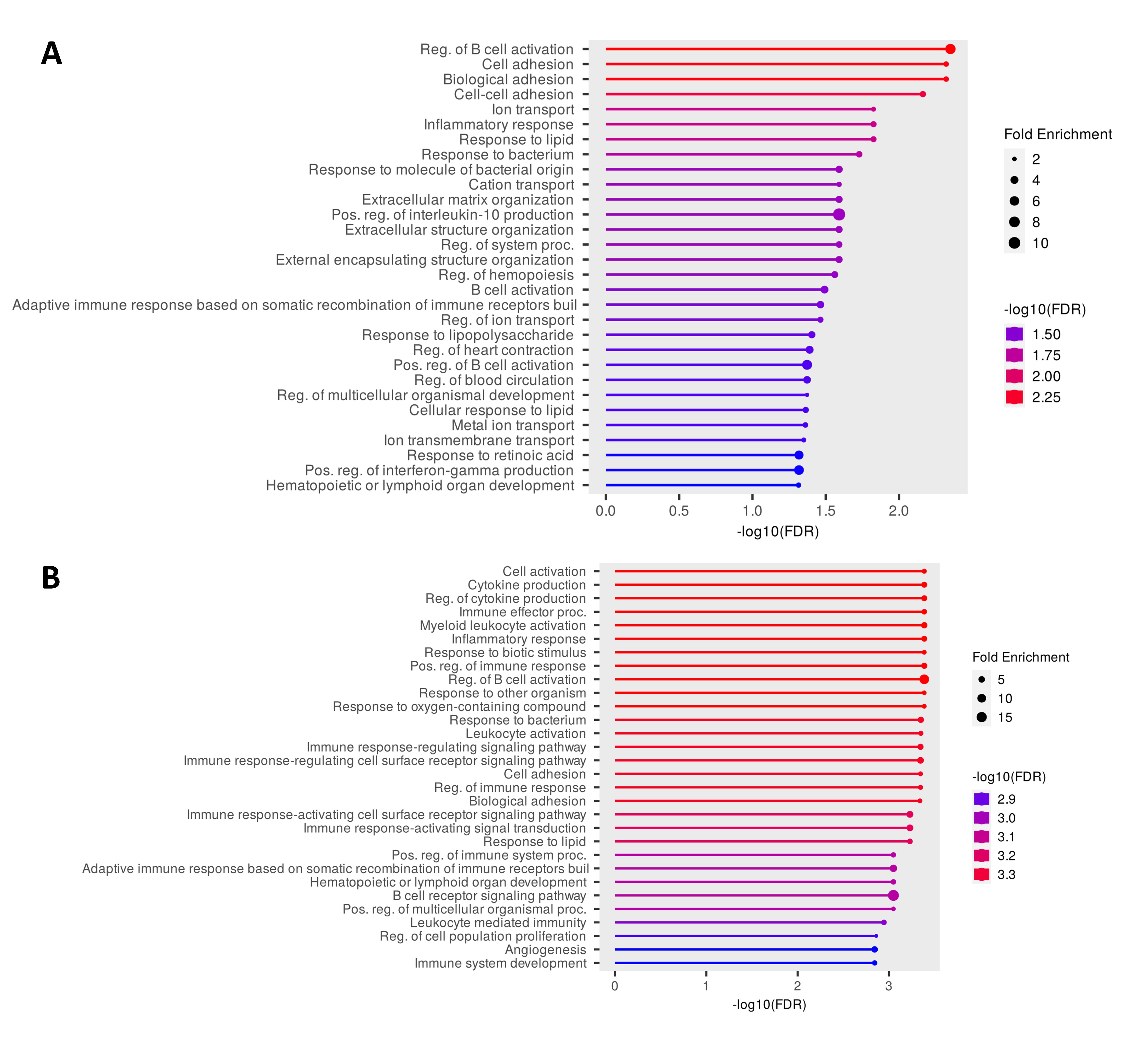

Supplement: Supplementary file 6 [file Image_4.jpeg]

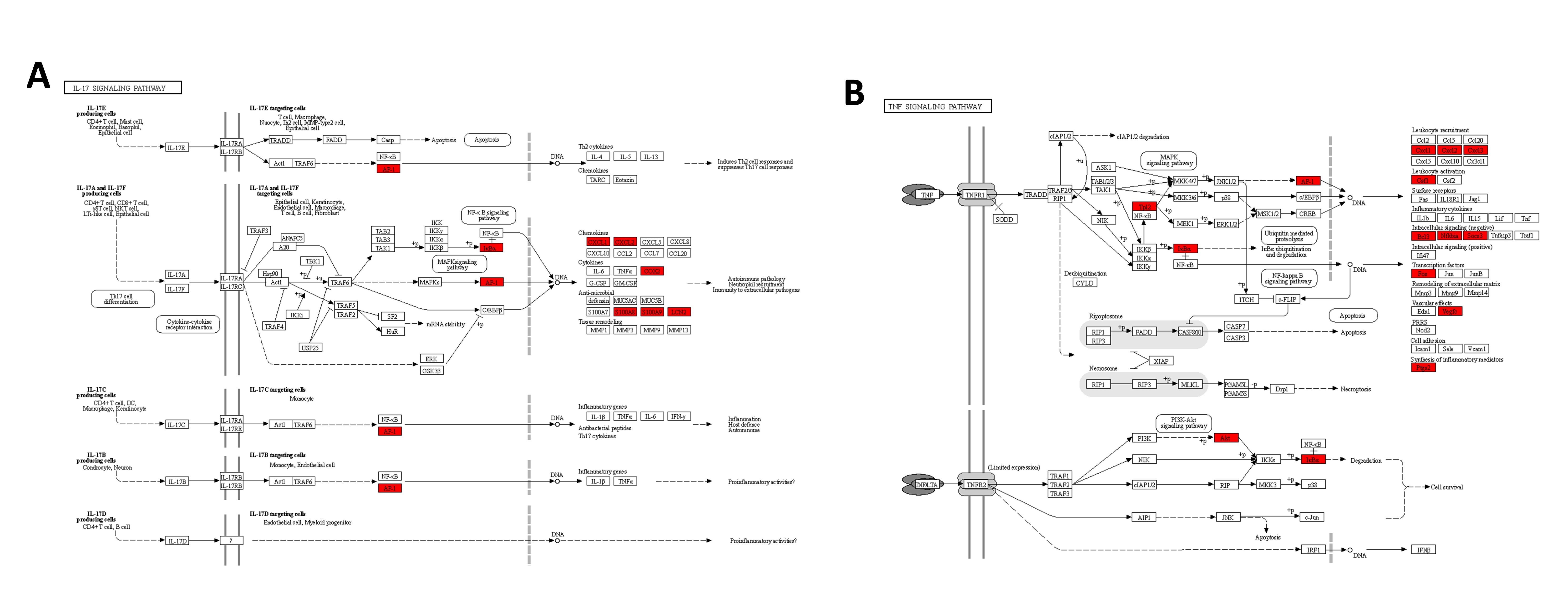

Supplement: Supplementary file 7 [file Image_5.jpeg]

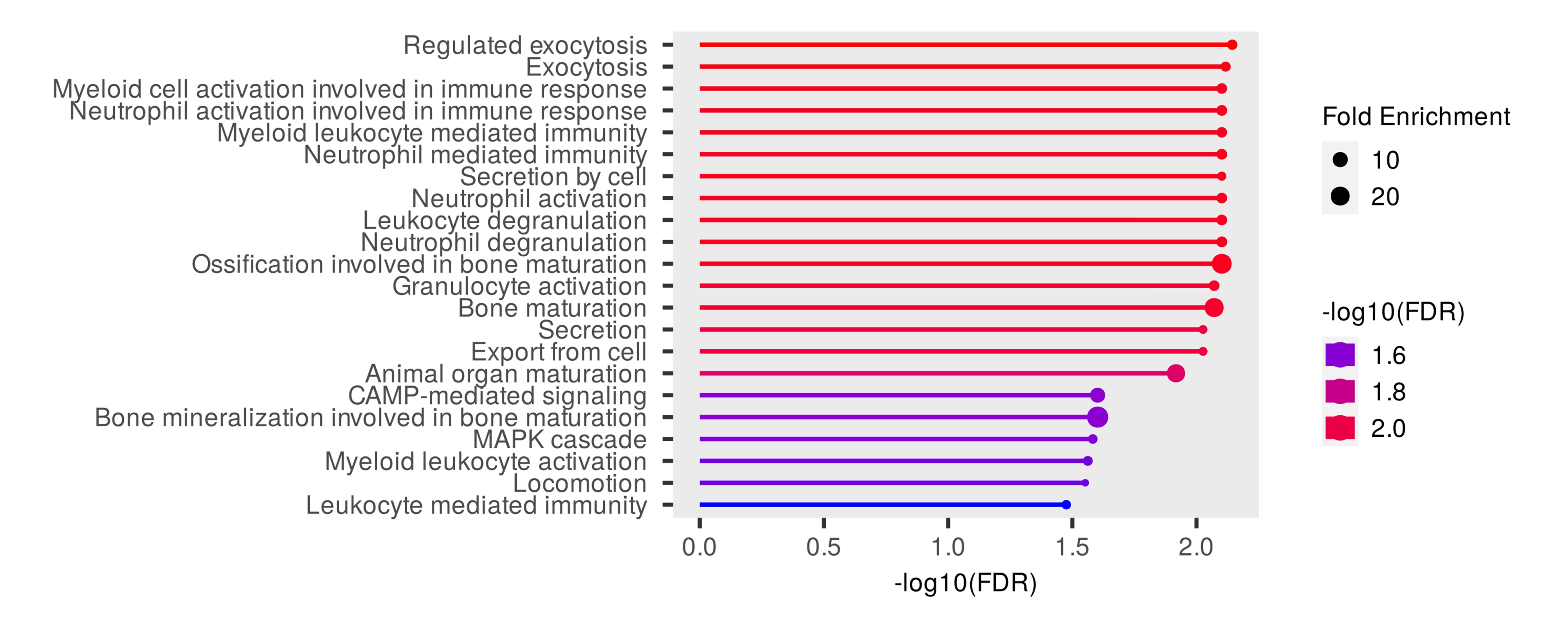

Supplement: Supplementary file 8 [file Image_6.jpeg]

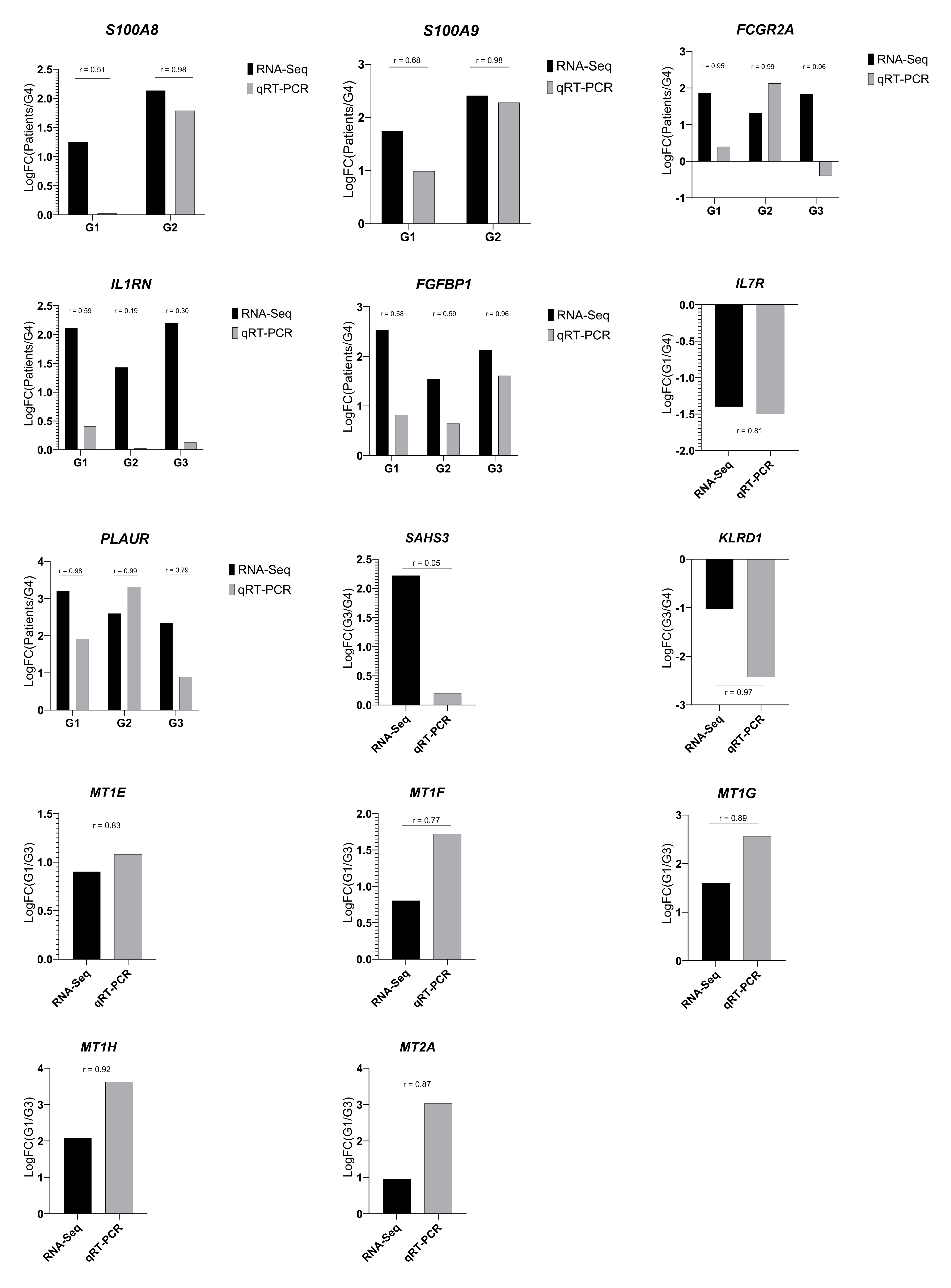

Supplement: Supplementary file 9 [file Image_7.jpeg]

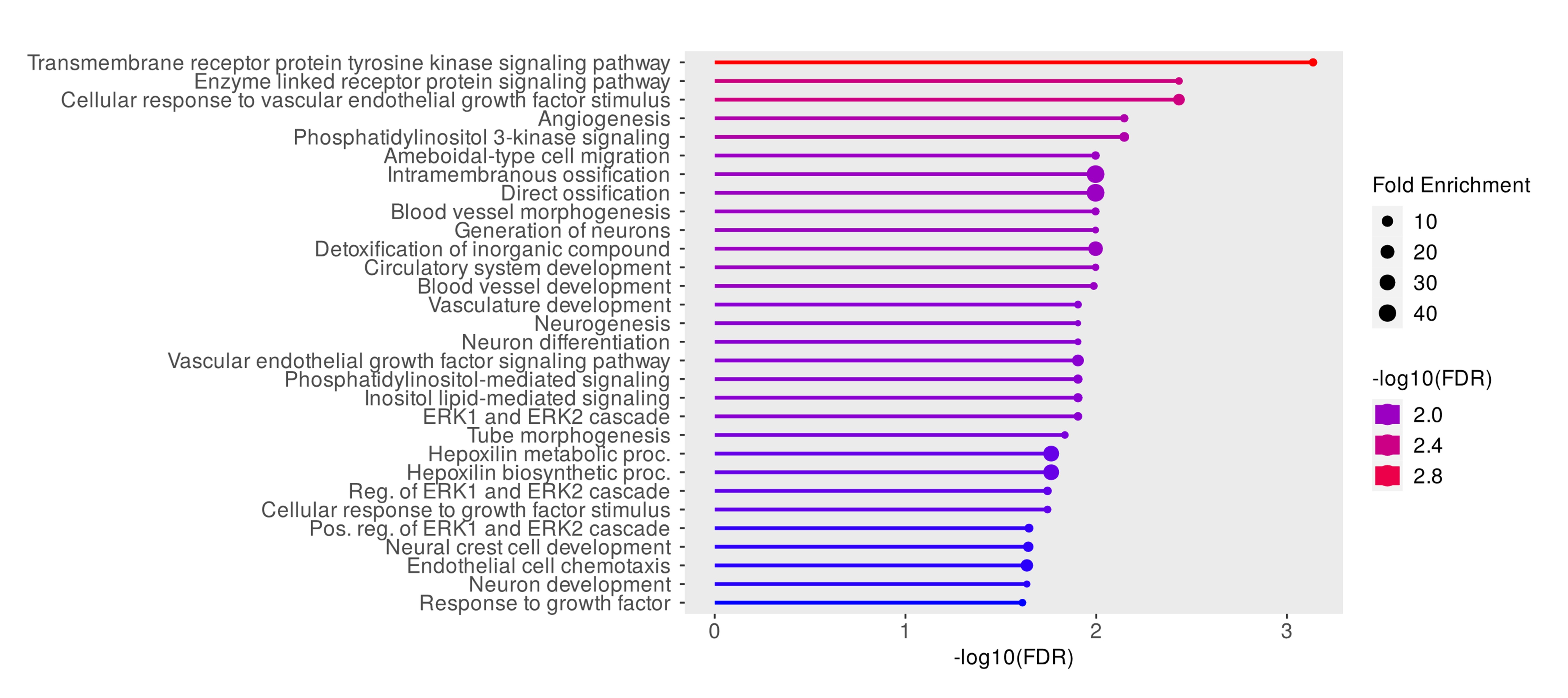

Supplement: Supplementary file 10 [file Image_8.jpeg]
